# Human social buffer in goats and dogs

**DOI:** 10.1007/s10071-024-01861-x

**Published:** 2024-02-14

**Authors:** Anna Scandurra, Biagio D’Aniello, Maria Elena Pero, Claudia Pinelli, Alfredo Di Lucrezia, Raffaella Tudisco, Piera Iommelli, Vincenzo Mastellone, Pietro Lombardi

**Affiliations:** 1 Department of Biology, University of Naples Federico II, Via Cinthia, 80126 Naples, Italy; 2 Department of Environmental, Biological and Pharmaceutical Sciences and Technologies, University of Campania “Luigi Vanvitelli”, Via Vivaldi, 43, 81100 Caserta, Italy; 3 Department of Veterinary Medicine and Animal Productions, University of Naples Federico II, Via Delpino 1, 80137 Naples, Italy; 4 Department of Pathology, Anatomy and Cell Biology, Columbia University, New York, 10032 USA

**Keywords:** Cortisol, Dog (*Canis lupus familiaris*), Domestication, Goat (*Capra hircus*), Stress

## Abstract

The primary goal of this study was to explore the social buffering effect that humans offer to goats and dogs with limited exposure to human socialization, particularly in situations involving interactions with unfamiliar humans. A total of 13 dogs and 14 goats were selected for the study, all of which had limited prior socialization with humans. Each animal was placed in a testing room with unfamiliar humans for 15 min. Three experimenters aimed to establish a comfortable environment, encouraging social interaction by offering food to the animals and assessing the animals’ willingness to accept food and their response to being approached and petted. If both conditions were satisfied, the animals were classified as “social”. If one or none of the conditions were met, the animals were classified as “not social”. Cortisol levels were measured by collecting blood samples before and after the test. Non-parametric tests together with a GzLM showed that the effect of human social buffering in goats was different in comparison to dogs: goats exhibited higher cortisol levels after the test, while dogs did not show a significant change. Further analysis demonstrated that “social” goats had a lower likelihood of experiencing significant changes in cortisol levels than dogs. Thus, once human interactions are accepted, both species could benefit from social buffering. In summary, this study enhances our understanding of how dogs and goats respond to social interactions with humans in the social buffering effect.

## Introduction

The social buffering effect has been widely documented in various vertebrate species (Hennessy et al. 2020). Extensive research has demonstrated that the mere presence of a conspecific social partner can effectively alleviate or diminish both physiological and behavioral responses to stressors, particularly regarding novelty-induced cortisol levels (Kikusui et al. 2006). One of the key mechanisms underlying the social buffering effect is the modulation of the hypothalamic–pituitary–adrenal (HPA) axis, which plays a vital role in regulating the release of stress hormones, including cortisol, in response to stressors. The presence of a social partner can significantly influence the functioning of the HPA axis in stressful situations, reducing cortisol secretion and restoring psycho-emotional equilibrium (von Holst 1998).

Beyond the intraspecific social buffering effect, it is intriguing to explore the interspecific social buffering effect, particularly in the context of interactions between humans and domesticated animals. This exploration is particularly noteworthy due to the targeted impact of the domestication process on changing the emotional responses of domesticated animals. Specifically, the process of domestication involved selective breeding and taming, which favored animals exhibiting diminished fear and aggression towards humans (Agnvall et al. 2018). As a consequence, emotional reactivity has been gradually reduced across subsequent generations (Diamond 2002). The social buffering effect provided by humans has been extensively studied in dogs. Numerous studies have indicated that dogs undergoing stressful situations can have a decrease in cortisol levels after engaging in interactions with humans (Coppola et al. 2006; Gunter et al. 2019; Hennessy 1997; Menor-Campos et al. 2011; Shiverdecker et al. 2013; Willen et al. 2017). Furthermore, studies have shown that the presence of a familiar person counteracts the natural increase in cortisol levels observed when dogs are placed alone in a novel environment (Tuber et al. 1996).

Dogs are believed to have been domesticated tens of thousands of years ago from wolf-like ancestors, and their closest extant relative is the grey wolf (Tancredi and Cardinali 2023; Thalmann et al. 2013; Vilà et al. 1997). Early humans may have observed the benefits of having wolves around, such as their keen senses, hunting skills, and ability to provide protection. Evidence from molecular and genetic data indicates that the separation of genetic lines leading towards dogs and modern wolves occurred well before the emergence of settled human communities in the Fertile Crescent. This suggests that the ancestors of dogs likely joined hunter-gatherer communities before the advent of settled agricultural practices (Tancredi and Cardinali 2023). As humans engaged in activities such as hunting, herding, and having valuables to be guarded, they likely selectively bred the friendliest and most cooperative wolves. This process favored traits that made dogs more compatible with human society, making them social companions (Miklósi et al. 2007), and enabling them to benefit from the human social buffering effect. However, when considering species domesticated for different purposes, such as livestock or agricultural animals, research on the social buffering effect and their response to humans may vary. We tested goats, which were primarily domesticated for their utility as a source of food (such as milk and meat) and other resources (such as fibres and skins), rather than for cooperative purposes (Nawroth 2017). Goats, as prey species, are expected to perceive humans as potential predators, and interactions with humans may trigger stressful responses. However, it is crucial to emphasize that interactions with humans can also be rewarding, such as receiving food, leading to positive emotional responses (Celozzi et al. 2022). Indeed, some studies have demonstrated that interactions between goats and humans can mitigate stressful responses in an intensive farming systems (Miller et al. 2018). This observation highlights the significance of studying the social buffering effect provided by humans in goats, making it a noteworthy aspect to further explore. Conducting such research would yield valuable insights into the dynamics of their relationship with humans and shed light on how it influences their overall welfare.

As a result, our current study aimed to examine the social buffering effect that humans can offer to goats by comparing the findings with a group of dogs. By doing so, we sought to gain a comparative understanding of how goats and dogs respond to and benefit from human social interactions, shedding light on the unique dynamics of their respective relationships with humans.

To examine the impact of domestication on the social buffering offered by humans, it is essential to control for potential confounding factors. It is necessary to eliminate known variables potentially affecting the social buffering effect above domestication, including human socialization and direct bonds with individuals involved in the testing procedure. However, achieving this goal is nearly impossible, as raising dogs and goats in total isolation from humans is both unavailable and unethical. Furthermore, there is evidence indicating that even minimal contact with humans induces significant changes in dog social behavior, particularly influencing the social buffering effect (Buttner et al. 2023). Despite these limitations, the comparison of species with similar levels of human socialization could still offer valuable comparative insights into how various domesticated species may alter emotional responses when interacting with unfamiliar humans. To accomplish this objective, we carefully selected groups of dogs and goats for the study, ensuring that they had limited prior socialization with humans. Moreover, we evaluated the animals in the presence of unfamiliar individuals to minimize any potential effects associated with previous interactions. Dogs and goats selected for the experiments were similarly raised and kept. Both species were allowed to leave their night-time enclosures during the day. In the case of goats, they were accompanied to pasture by their owner or an attendant in the morning, enabling them to interact with other goats. The dogs could remain in small groups freely around the farm. While this arrangement facilitated intraspecific socialization in both species, the opportunities for interspecific interaction, including human socialization, were limited. Human contact was kept at a minimal level for both dogs and goats and primarily involved a single caretaker who provided daily cleaning and feeding.

By comparing the social buffering effects in dogs and goats under these specific conditions, we aim to gain a better understanding of how different domesticated species with equally minimal human interaction history respond to social interactions with humans and whether similar social buffering mechanisms exist across species. Considering the cooperative nature of wolves' domestication and the ability of dogs to derive social buffering benefits even with minimal contact with humans (Buttner et al. 2023), it is anticipated that dogs would likely experience greater advantages from human-provided social buffering compared to goats, which have not been specifically selected for cooperative tasks.

## Material and methods

### Animals

In our study, we examined a total of 13 dogs (*Canis lupus familiaris*) with a mean age of 5 ± 1.75 years and 14 goats (*Capra hircus*) with a mean age of 2.79 ± 1.42 years. All dogs belonged to the Australian Cattle Dog breed, while all goats belonged to the *Camosciata Alpina* breed. All animals were females. The study was conducted at a farm called “Eugenia Palumbo's Funky Farm” located in Cassino, Italy. Both dogs and goats were raised in enclosures on the farm. The dogs were hosted in nocturnal recovers in groups of 2–3, while the goats were kept all together. The goats were allowed to leave the pens for grazing activities in the morning for around 4 h. The dogs had daily access to leave the kennel and freely interact between them in small groups for approximately 1 h during the day. Interactions between the dogs and goats were limited, and the level of human socialization was also limited. The animals only encountered humans during routine care activities and periodic veterinary check-ups. The goats were additionally handled for milking. Crucially, it should be noted that both the dogs and goats included in the study were born and raised on the farm itself.

### Testing procedure

The experiments were conducted within a rectangular room unknown to the goat and dog subjects, measuring around 30 m^2^, scheduled during the afternoon in the same time slot to prevent potential bias linked to the circadian cortisol cycle (Giannetto et al. 2014). The room was equipped with two cameras (Sony^®^ HDR CX115 and Sony^®^ HDR-PJ260VE) strategically positioned in separate corners. To ensure consistency and control over lighting conditions and to avoid visual disturbances, cardboard covered the windows located on two opposite sides of the room. Between one animal test and another, the room was cleaned using a mild-scented detergent. Each animal was accompanied into the room by the caregiver and left there for a duration of 15 min, along with three human strangers (i.e., one man and two women). Throughout the testing procedure, the people created a friendly atmosphere and attempted to engage in contact with the animals. The human experimenters positioned themselves strategically within the room to allow for natural interactions with the animals. They varied their positions to observe how the animals responded in different situations. The experimenters aimed to establish a comfortable environment, and they encouraged social interaction. Specifically, the experimenter assessed whether the animals accepted food by hand after placing food on the floor and whether the animals approached and accepted petting. If both conditions were met, the animals were classified as “social”. If one or none of the conditions were met, the animals were classified as “not social”.

### *Blood sampling and cortisol assay*

All blood samples were collected from the jugular and cephalic veins of goats and dogs, respectively. The blood sample collection was performed by two of the authors of the study, who are veterinarians. Serum samples were collected in serum vacutainer brand tubes (Beckton Dickinson Vacutainer Systems, UK), centrifuged for 15 min at 1500×*g* to obtain the serum that was aliquoted, and immediately stored at − 20 °C thereafter. Cortisol concentrations were analyzed as a single batch using a solid-phase, competitive chemiluminescent enzyme immunoassay (Immulite^®^ 2000, Siemens).

Cortisol levels were measured in blood samples collected both before (*T*0) and after (*T*1) the test. The blood samples were collected immediately after they exited the enclosures, ensuring a time frame of less than 2 min from the initiation of the testing procedure. This timing was chosen based on previous studies in rats, where it was demonstrated that such a rapid blood sampling protocol minimizes any potential impact on cortisol levels (Coover et al. 1979). Moreover, the time interval between each sampling and the subsequent one was kept under 20 min. Previous research conducted on dogs has indicated that even just 15 min of human interaction is adequate to elicit a cortisol response (Willen et al. 2017).

### Statistical approach

The statistical approach employed in the study allowed for a direct comparison between the two species. Considering the limitations posed by the small sample size, non-parametric statistical tests were chosen, as they provide a robust analysis without relying on assumptions about the data distribution. Specifically, the Mann–Whitney *U* test was utilized to compare the differences in cortisol levels between species, while the Wilcoxon test was used to explore variations in cortisol levels within each species before and after the test. Similarly, when comparing social and not social subjects, the same non-parametric approach was followed. In this case, the subjects categorized as “social” and “not social” within each species (dogs and goats) were combined/pooled together. This was necessary because the small sample size within each species did not provide enough statistical power to analyze them separately in non-parametrical tests. To further control for the results obtained from the non-parametric tests and to examine the effect of age, a Generalized Linear Model (GzLM) was utilized. In this model, the response variable was the variation of cortisol (delta cortisol), which was not normally distributed. The explanatory factors considered were species and sociability, while age was included as a covariate. The primary objective of the GzLM was to assess the main effects of the factors (species and sociability), as well as the covariates (age). Additionally, the first level of interaction between species and sociability was tested to evaluate potential combined effects.

## Results

### Interspecies comparison

**Fig. 1:**
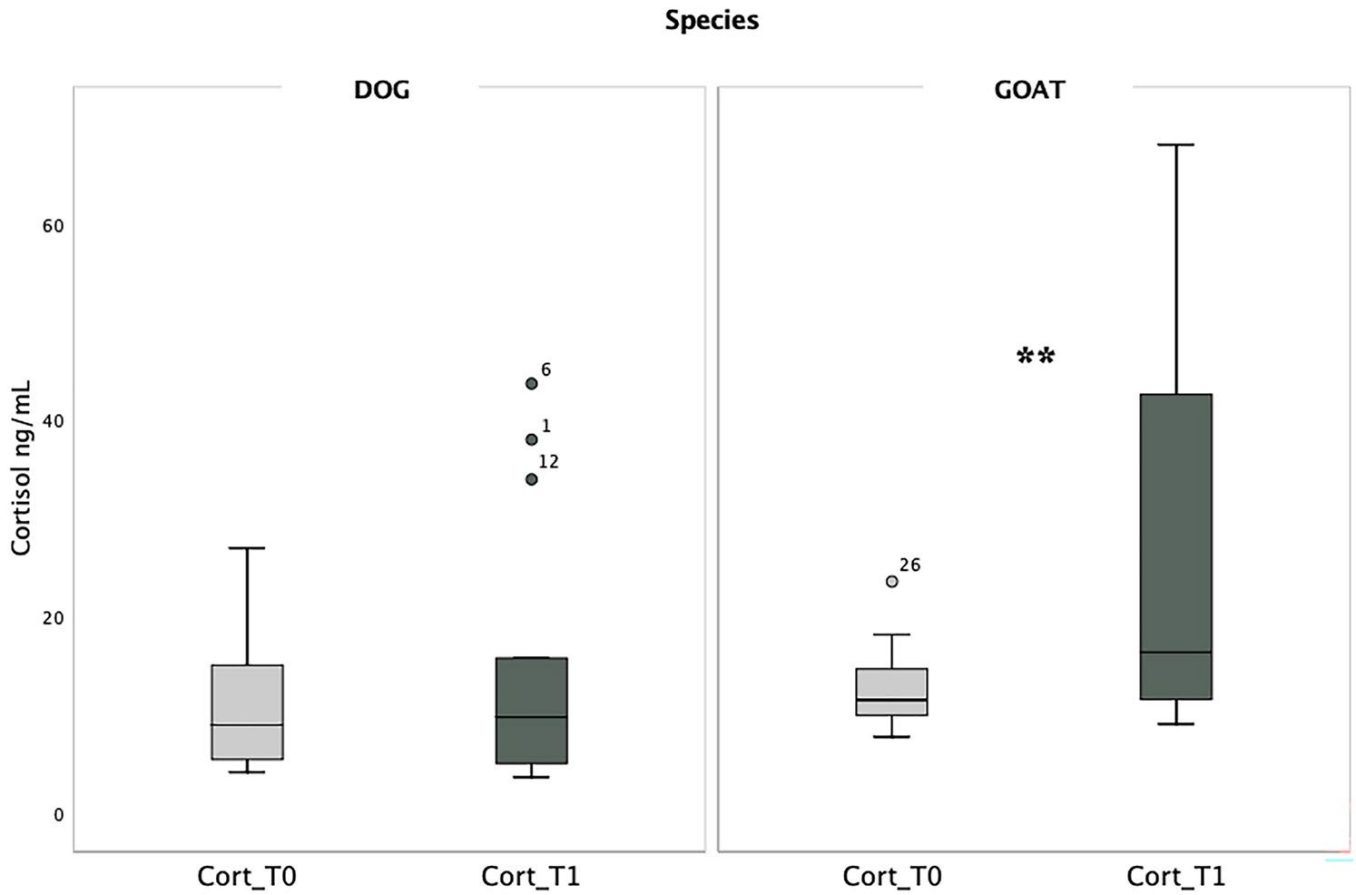
Cortisol levels in dogs (left) and goats (right) expressed in ng/mL before (T0) and after (T1) the test. Thick black lines: medians; boxes: interquartile range (from 25 to 75th percentile); thin vertical lines: minimum and maximum values. ** *p* < 0.01. The numbers 1, 6, 12, 26 within the figure correspond to the subject ID

Before the test (*T*0), the median cortisol levels in goats were higher (median = 11.55 ng/mL) compared to dogs (median = 9.00 ng/mL), but the difference was not statistically significant (Z = −1.408, *p* = 0.159). However, after the test (*T*1), the difference in cortisol levels became significantly higher. The median cortisol level in goats was 16.40 ng/mL, while in dogs 9.80 ng/mL (*Z* = −2.112, *p* = 0.035; Fig. 1).

Within each species, the within-subjects comparison showed that there was a significant increase in cortisol levels in goats after the testing procedure (*Z* = −2.638, *p* = 0.008). However, in dogs, there was no significant change in cortisol levels (*Z* = −0.874, *p* = 0.382; Fig. 1).

### *“Social”*–*“not social” comparison*

**Fig. 2:**
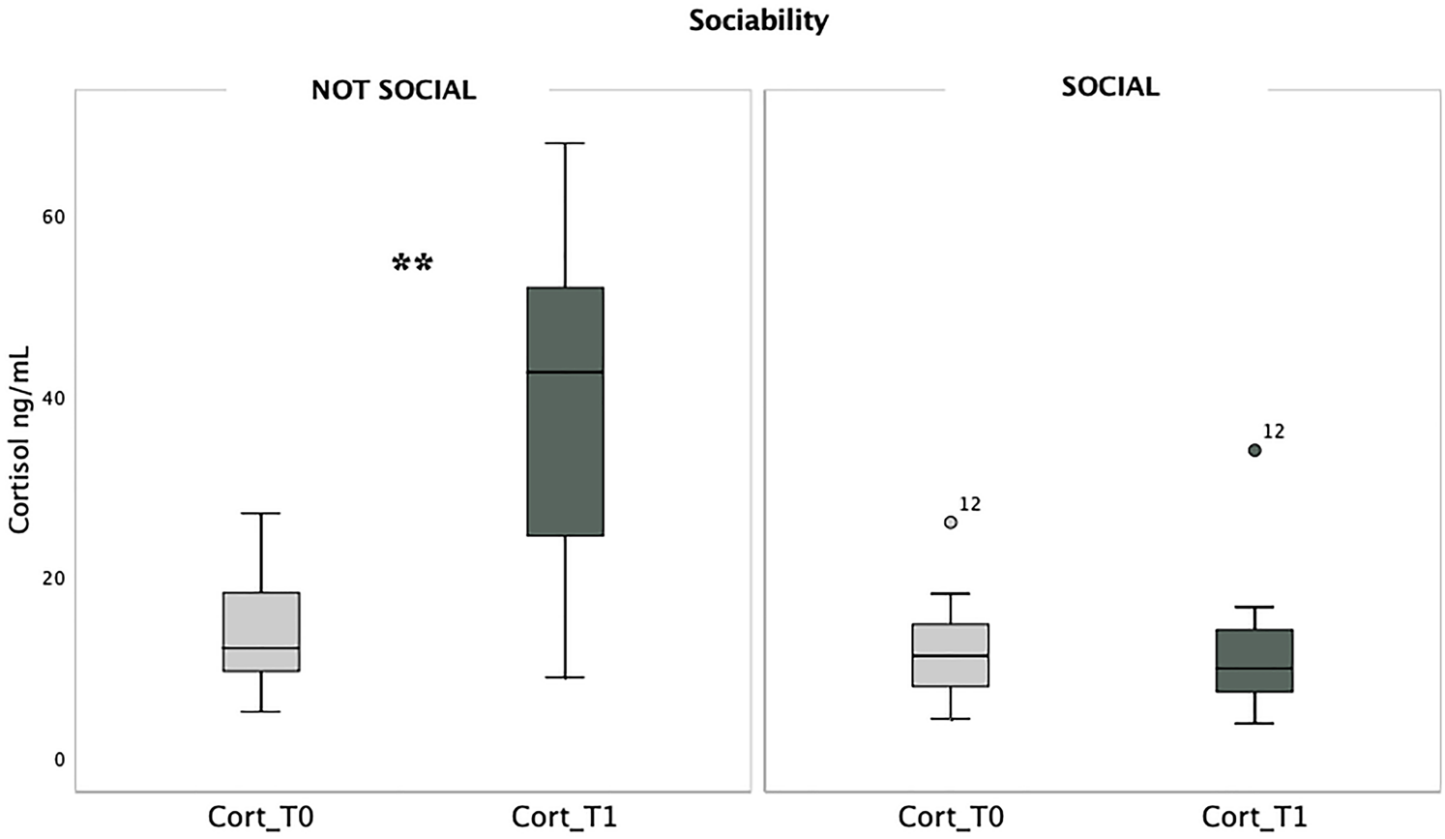
Cortisol levels expressed in ng/mL in NS (i.e., not social group, on the left) and S (i.e., social group, on the right) before (T0) and after (T1) the test. Thick black lines: medians; boxes: interquartile range (from 25 to 75th percentile); thin vertical lines: minimum and maximum values. **p < 0.01. The number 12 within the figure corresponds to the subject ID

Among the experimental subjects, 10 individuals (4 dogs and 6 goats) refused any form of social interaction with humans. Mann–Whitney pairwise comparison to examine cortisol levels between the groups that had not social (NS) interaction and those that had social (S) interaction showed that at T0, cortisol levels were found to be similar between the NS and S groups. The median cortisol level for the NS group was 12.05 ng/mL, while it was 11.20 ng/mL for the S group. The statistical analysis indicated that there was no significant difference between the two groups at this time point (*Z* = −0.929, *p* = 0.353; Fig. 2). However, at T1 the NS group exhibited higher cortisol levels compared to the S group. The median cortisol level for the NS group at T1 was 42.60 ng/mL, whereas it was 9.80 ng/mL for the S group. The statistical analysis using the Mann–Whitney test revealed a significant difference between the groups (*Z* = −3.265, *p* = 0.001; Fig. 2).

Furthermore, the Wilcoxon test showed a significant increase in cortisol levels within the NS group after the test (*Z* = −2.803, *p* = 0.005), while there was no significant variation in cortisol levels within the S group (*Z* = −0.379, *p* = 0.705).

### Generalized linear model

The Generalized Linear Model (GzLM) was utilized in this study, with the change in cortisol (ΔC) as the response variable. The full model’s likelihood ratio chi-squared test demonstrated a significant improvement in fit compared to the null model (Omnibus Test: *χ*^2^ = 29.490, *p* < 0.001). The analysis revealed several significant effects. First, there was a positive main effect of species (*β* = 18.883; *χ*^2^ = 7.019; *p* = 0.008), indicating that species had an impact on the probability of experiencing an increase in ΔC after the test. Specifically, goats had a higher probability of exhibiting an enlarged ΔC compared to dogs. Second, a negative main effect of sociability was observed (*β* = −13.665; *χ*^2^ = 5.531; *p* = 0.019). This suggests that subjects who accepted human interaction had a lower probability of experiencing variation in ΔC. Additionally, a negative interaction effect between goats and sociability was found (*β* = −18.547; *χ*^2^ = 5.524; *p* = 0.019). This interaction implies that goats, specifically, had a lower probability of experiencing changes in ΔC after accepting human contact. The combination of being a goat and having a higher social tendency resulted in a reduced likelihood of ΔC variation. Notably, no main effects of age were found to be significant in this analysis, indicating that age did not significantly influence the probability of ΔC variation.

## Discussion

In this study, it was observed that goats had higher cortisol levels before the testing procedure compared to dogs, but the difference was not statistically significant. However, it is important to note that the lack of statistical significance in the cortisol difference may be attributed to the small sample size and the presence of outliers, which can introduce internal variability and affect the results. The limited number of subjects within each species could have made it difficult to detect significant differences in cortisol levels, even if they exist. Nevertheless, it is worth mentioning that any potential differences in cortisol levels before treatment are likely related to the inherent physiological variations between species. These differences in cortisol levels before the test do not provide meaningful insights on their own, as they are expected due to the distinct physiology of each species (Kannan et al. 2000; Mooney and Peterson 2004). Therefore, the focus of the study should primarily be on the changes observed after the testing procedure rather than before.

The results obtained from the non-parametric tests were consistent with those of the GzLM analysis, indicating a significant impact of the testing procedure on cortisol levels in goats. This effect resulted in an overall increase in cortisol levels within the goat group. However, no significant change was observed in dogs. These findings in dogs align with previous studies on the isolation paradigm, which demonstrate the positive influence of human-provided social buffering. Research has shown that the presence of people can help mitigate the rise in cortisol levels when dogs are isolated in an unfamiliar environment (Tuber et al. 1996). However, it appears that this psycho-emotional process of social buffering mediated by humans is ineffective in goats. Taking into account the comparable ontogenetic trajectory of the goats and dogs included in this study, it appears that the domestication process has contributed to the observed disparities. It is plausible that selection for cooperative work and companionship in dogs has fostered an emotional bond with humans, facilitating the activation of social buffering mechanisms. Conversely, goats, primarily domesticated for utilitarian purposes such as food and resource production, may not have developed a comparable level of emotional association with humans. Alternatively, the minimal level of socialization with humans within our experimental sample may have been enough to utilize humans as coping mechanisms in dogs (Buttner et al. 2023) but not in goats. Based on these findings, it can be concluded that the hypothesis proposed in this study has been supported. On the other hand, it is important to consider the distinct behavioral ecology of the species. While both goats and dogs are social animals that live in groups, separation from the pack is more typical for dogs. They are often observed wandering alone (Boitani and Ciucci 1995; Majumder et al. 2014; Pal et al. 1998) or periodically splitting from the group as part of a fission–fusion mechanism (Finzi et al. 2023). In contrast, such behavior is not commonly observed in goats, as living outside the group exposes them to a higher risk of predation. Therefore, dogs may be more accustomed to separation from the pack and exhibit lower levels of stress compared to goats, which in turn could have affected our outcomes. Further studies are necessary to investigate this hypothesis.

Our findings also show that the subjects who refused social interaction with humans had comparable cortisol levels to those who accepted social interaction during the test. After the test, the cortisol levels of the former group significantly increased, whereas the cortisol levels of the latter group did not exhibit a significant change. The GzLM analysis results confirm the main effect of species and sociability in predicting the likelihood of cortisol level changes. However, goats had a lower probability of experiencing cortisol changes than dogs after accepting human contact. This means that once goats accept human interaction, goats could benefit from social buffering better than dogs. Overall, the findings indicate that although goats as a group are more likely to experience an increase in cortisol levels, this outcome was primarily driven by subjects who refused human interaction. Therefore, the key factor in benefiting from social buffering was not simply the presence of people but rather an active interaction with them. Indeed, research has shown that physical contact with humans has beneficial effects on goats (Leite et al. 2020).

The current data do not provide evidence regarding the reasons why some subjects of both species encountered difficulties interacting with humans. However, it is well-known that testing goats in ethological procedures can pose challenges in terms of their interaction with humans. However, these challenges can be overcome by implementing a specific acclimation protocol (Langbein et al. 2018; Mastellone et al. 2020; Nawroth 2017), which was not the case in the current research.

While our study provides valuable insights into the social buffering effects in goats and dogs, it is important to acknowledge certain limitations. First, the study's sample size was relatively small, consisting of 13 dogs and 14 goats, all of which were females. This limited sample size, along with the exclusive inclusion of female subjects, may impact the generalizability of our findings. We recognize the potential influence of sex-related variables on stress responses. In specific contexts, particularly within cooperative environments, females might display more prosocial behaviors towards unfamiliar individuals in contrast to males (Lore and Eisenberg 1986; Persson et al. 2015; Scandurra et al. 2018). Considering this distinction, it is possible that males might potentially encounter distinct benefits arising from the social buffering effects provided by interactions with humans. Therefore, expanding the study to include male subjects could provide a more comprehensive understanding of the social buffering effect across sexes. Second, this study specifically targets groups of dogs and goats with limited human socialization. Consequently, additional research encompassing a more varied range of dog or goat populations with different socialization degrees is recommended to broaden the generalizability of our findings.

In conclusion, the study provides support for the hypothesis by demonstrating the contrasting effects of social buffering in goats and dogs. The findings indicate that as a group, goats did not benefit from the social buffering provided by humans. However, it is important to note that once goats accepted social interaction with humans, they could benefit from the social buffering effect. This suggests that active engagement with humans is crucial for reaping the benefits of social buffering in both goats and dogs. These results shed light on the differential responses of species to human interaction and emphasize the significance of considering individual factors, such as acceptance of social interaction, in understanding the impact of social buffering. Further research is warranted to delve deeper into the underlying mechanisms and explore ways to enhance social interaction and well-being in both goats and dogs.

## Data Availability

The dataset is available from the authors upon reasonable request.
